# Endovascular recanalization of acute ischemic stroke patients exhibiting large vessel occlusion after pulmonary lobectomy: case series

**DOI:** 10.1186/s12883-022-02866-0

**Published:** 2022-09-12

**Authors:** Renjie Ji, Ziqi Xu, Hanfeng Chen, Benyan Luo

**Affiliations:** Department of Neurology, The First Affiliated Hospital, School of Medicine, Zhejiang University, Hangzhou, 310003 China

**Keywords:** Endovascular recanalization, Ischemic stroke, Pulmonary lobectomy

## Abstract

**Objective:**

We analyzed the outcomes of patients suffering acute ischemic stroke (AIS) with large vessel occlusion (LVO) soon after pulmonary lobectomy.

**Methods:**

We retrospectively reviewed the clinical records of patients who underwent pulmonary lobectomy to treat primary lung cancer. We retrieved clinical characteristics and the incidence of AIS with LVO. The clinical courses of patients who experienced AIS were reviewed.

**Results:**

In 10 (0.3%) of 3406 patients, AIS with LVO developed soon (within 3 days) after pulmonary lobectomy. The lung resection site was on the left in eight patients (80%). All patients underwent thrombectomy and achieved complete recanalization (Thrombolysis in Cerebral Infarction [TICI] 3). The average time between symptom onset and recanalization was 165.5 min. Nine (90%) patients exhibited favorable outcomes (modified Rankin scale [mRS] score ≤ 2) at the 3-month follow-up.

**Conclusion:**

Endovascular therapy effectively treats AIS with LVO that develops after lung surgery, and direct aspiration is a promising strategy. A large, multicenter study is warranted to further confirm these findings.

## Introduction

Perioperative acute ischemic stroke (AIS) is uncommon in patients undergoing pulmonary lobectomy/pneumonectomy; the incidence is approximately 0.4–0.6% [1, 2]. The mortality rate after a first stroke is approximately 15% in the general population and 26% when stroke occurs perioperatively [3]. The neurological deficits after AIS impose heavy burdens on patients, families, and society.

The possible causes of IS caused by pulmonary lobectomy include a thrombus derived from a pulmonary vein stump (PVS) [4], a thrombus caused by perioperative atrial fibrillation, and low-level perfusion during operation. It is thought that (particularly, left) PVS thrombosis may be the most common mechanism [5].

Data on AIS after lobectomy are limited to case reports; an in-depth understanding and a management consensus are lacking. We reviewed the clinical data of AIS patients with large vessel occlusions (LVOs) that developed after lobectomy. We discuss the possible pathogenesis and treatment options.

## Patients and methods

This retrospective study was performed at our hospital, which has a comprehensive stroke center. We gathered data from September 2019 to October 2021. Patients who underwent endovascular treatment after pulmonary lobectomy were included. All patients underwent video-assisted thoracic surgery pulmonary lobectomy. The clinical characteristics of all patients and data on the surgical procedures of lobectomy and endovascular recanalization were collected through the electronic medical records system of our hospital. Continuous variables are summarized as the mean and standard deviation. Statistics were performed using SPSS Statistics 21 (IBM Inc., USA). This study was approved by the clinical research ethics committee of the First Affiliated Hospital of Zhejiang University (Reference number: 2021IIT No. 963), and all patients gave written informed consent.

### Diagnosis and evaluation of AIS

AIS is defined as sudden neurologic dysfunction caused by focal brain ischemia lasting more than 24 hours or with evidence of acute infarction on brain imaging, irrespective of symptom duration [6]. Multimodal brain computed tomography (CT) (noncontrast CT; CT angiography [CTA]; and CT perfusion [CTP]) was performed to locate LVOs because intravenous thrombolysis is contraindicated in the perioperative period.

## Results

From September 2019 to October 2021, the total number of patients who underwent pulmonary lobectomy was 3406. Of the 3406 patients, 10 patients (three males and seven females) developed AIS with LVO after lobectomy. Eight (80%) patients had undergone left-sided surgery; patient age ranged from 42 to 79 years (average 60.3 years) (Table 1). Cerebral infarction developed from postoperative hours 7–72 (average 34.2 ± 25.6 h). The lobectomy operation time was 51–170 min, and the blood loss was 10–50 mL (average 27.5 ± 18.4 mL); all pulmonary veins were divided using a stapler.

**Table 1:** Clinical and surgical characteristics (pulmonary lobectomy) of patients with postoperative cerebral infarction

Case	Age	Sex	Preoperative complication	Location of lobectomy	Pulmonary stump treatment	Operation time (min)	Blood loss (ml)	Pathology	Postoperative AF
1	48	F	None	RLL	Taper	75	20	adenocarcinoma	None
2	68	M	None	RUL	Taper	51	10	adenocarcinoma	None
3	54	M	Hypertension	LLL	Taper	120	50	adenosquamous carcinoma	None
4	79	F	CHD	LLL	Taper	80	15	adenocarcinoma	None
5	67	F	None	LUL	Taper	170	60	adenocarcinoma	None
6	61	F	None	LLL	Taper	60	20	adenocarcinoma	None
7	42	F	None	LUL	Taper	74	50	adenocarcinoma	None
8	58	M	None	LLL	Taper	64	20	adenocarcinoma	None
9	57	F	None	LLL	Taper	70	20	adenocarcinoma	None
10	69	F	Ischemic stroke	LUL	Taper	56	10	adenocarcinoma	None

*CHD* coronary heart disease, *LUL* left upper lobectomy, *LLL* left lower lobectomy, *RUL* right upper lobectomy, *RLL* right lower lobectomy

**Fig. 1:**
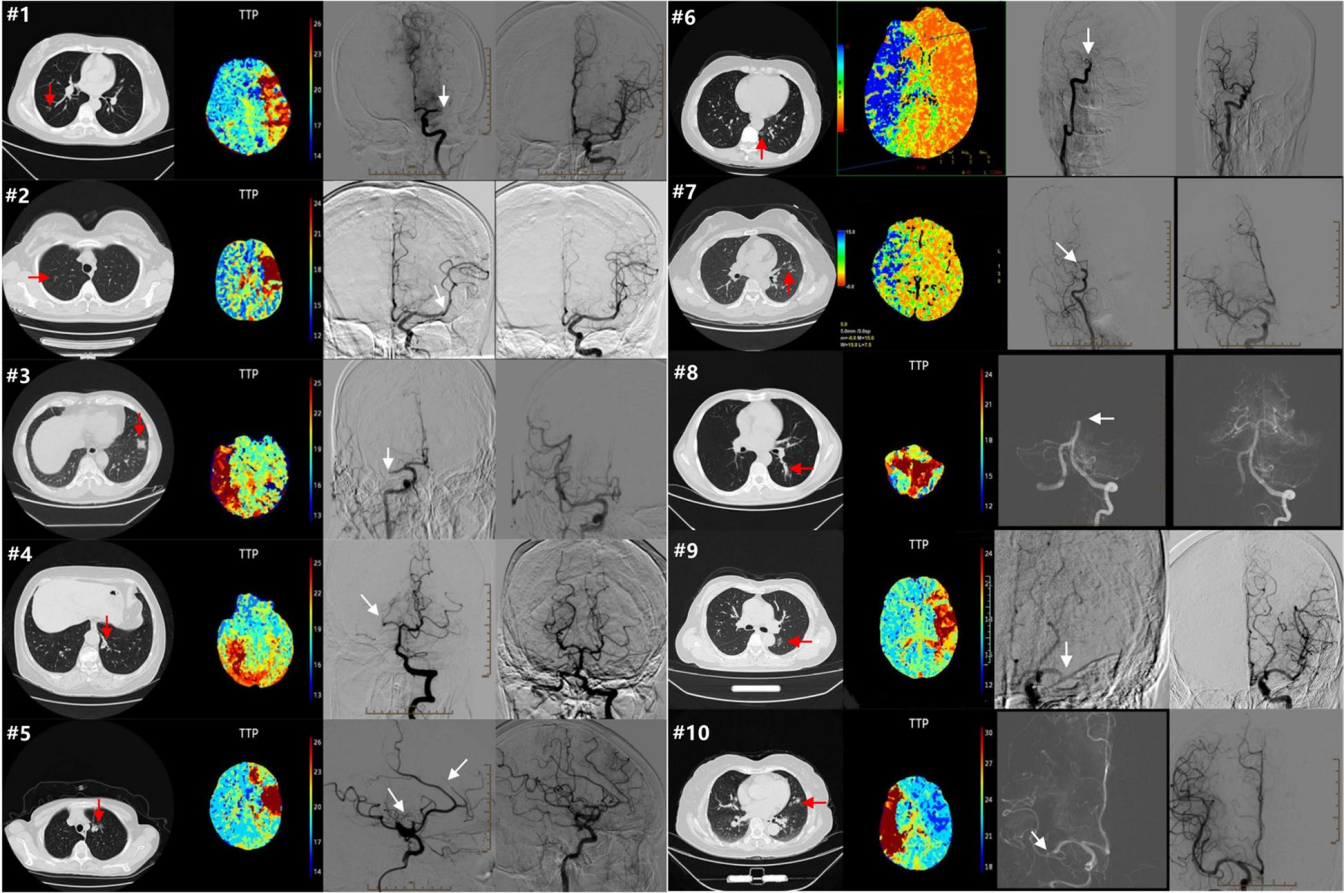
Medical images in 10 patients (location of lesion on Lung CT (red arrowheads), CT perfusion, location of the occlusive artery on DSA (white arrowheads))

The occluded arteries were the right middle cerebral artery in three patients (including one with right MCA and ACA), the left middle cerebral artery in three patients, the distal right internal cerebral artery in two patients, the basal artery in one patient, and the right posterior cerebral artery in one patient (Table 2). All patients underwent emergency endovascular therapy delivered by interventional neuroradiologists; all achieved successful recanalization and had a thrombolysis in cerebral infarction (TICI) reperfusion grade of 3 (Fig. 1). All patients underwent local anesthesia or conscious sedation (not general anesthesia). In our patients, the average postoperative time to symptom onset was 34.2 h, and the average time from onset to puncture was 129 min. The time between symptom onset and recanalization ranged from 120 to 220 min (average 165.5 ± 37.6 min). The average endovascular therapy duration was 36.5 (±6.7) min. Nine (90%) patients experienced favorable outcomes (modified Rankin scale [mRS] score ≤ 2) at the 3-month follow-up; one patient (No. 5) developed hemiplegia of the left limbs (mRS = 4). All patients were given anticoagulation therapy (rivaroxaban 20 mg per day) after the endovascular operation after imaging (brain CT or magnetic resonance imaging) revealed no hemorrhage. No ischemic stroke recurrence was noted during later follow-up (mean 13.7 months).

**Table 2:** Summary of acute phase cerebral infarction patients with endovascular theray after pulmonary lobectomy

Case	Interval^a^ (h)	Location of Thromboembolism	NHISS score	Time to puncture (min)	Time to recanalization (min)	EVT method	mRS of 3 month
1	7	Left MCA (M1)	12	120	150	CA	1
2	5	Left MCA (M2)	10	120	160	CA	1
3	45	Right MCA (M1)	14	180	220	CA	1
4	24	Right PCA (P2)	10	180	210	SR	2
5	73	Right MCA (M2) and ACA (A2)	12	120	160	SR	4
6	12	Right ICA (C7)	13	100	135	CA	0
7	48	Right ICA (C7)	11	180	220	CA	1
8	12	BA	18	120	150	CA	0
9	70	Left MCA (M1)	13	80	130	CA	1
10	46	Right MCA (M1)	10	90	120	CA	1

Interval ^a^: from operation to the occurrence of cerebral infarction

*MCA* middle cerebral artery, *PCA* posterior cerebral artery, *ACA* anterior cerebral artery, *CA* contact aspiration, *SR* stent retriever

## Discussion

### Mechanism of cerebral embolism development after pulmonary lobectomy

Postoperative atrial fibrillation (POAF) is a common complication in patients undergoing lung surgery [7, 8] (approximately 14–20% [9, 10]). The cause remains unclear, but denervation caused by anatomical pneumonectomy and stress-mediated neurohumoral responses to anatomical pulmonary resection may cause AF in susceptible patients [11, 12]. POAF may increase the hospitalization time and the risk for perioperative cerebral embolism. However, none of our patients had evidence of paroxysmal AF during intraoperative and postoperative ECG monitoring.

Thrombosis in a PVS is a risk factor for cerebral embolism after pulmonary lobectomy. The PV differs from other veins in that it is directly connected to the left cardiac system. This may explain the high risk for cerebral embolism in patients with PVSs. The stump length affects the thrombosis risk after pulmonary lobectomy; the longer the stump is, the higher the risk. Of all patients undergoing pulmonary lobectomy, 3.3–3.6% developed PVS thrombosis; the figure reached 13.5–17.9% in patients with left PVSs [4, 13]. As the stump of the left superior PV is long, blood may stagnate and then become turbulent, increasing the risk for thrombosis. Of patients undergoing pulmonary lobectomy, the proportion of cerebral embolisms in those receiving left and left upper lobectomies was significantly higher than that in other patients, perhaps reflecting the longer stump of the left superior PV [5]. It has been suggested that the PVS should be shortened to prevent lobectomy-induced cerebral embolism. However, the PV must then be amputated near the pericardium, which can cause cardiac tamponade (a more critical complication than a cerebral embolism [14, 15];). Of our patients, 80% with cerebral embolisms had undergone left lobectomy; this proportion is similar to that in the literature. As thrombosis in a PVS can cause embolic events, we suggest that such patients should receive anticoagulants, particularly those undergoing left upper lobectomy or left pneumonectomy. However, bleeding complications after lobectomy may lead to serious consequences. It is controversial to suggest that all patients undergoing lobectomy should receive anticoagulants to prevent cerebral embolism [1]. Anticoagulant therapy is considered for high-risk patients. These patients may be selected according to the CHADS2/CHADS2 VASc score [16, 17], which is a criterion for cardiogenic cerebral embolism. This score may also be used to predict the risk of cerebral embolism after lobectomy, as blood congestion is also associated with it. We recommend that all patients should undergo contrast-enhanced CT after surgery to exclude PVS thrombosis. Furthermore, the examination should be performed in the very early phase after lobectomy, as postoperative ischemic stroke usually occurs immediately after pulmonary resection. Early detection of the thrombus may contribute to the prevention of postoperative ischemic stroke. If thrombosis is present, anticoagulation therapy should be added.

**Fig. 2:**
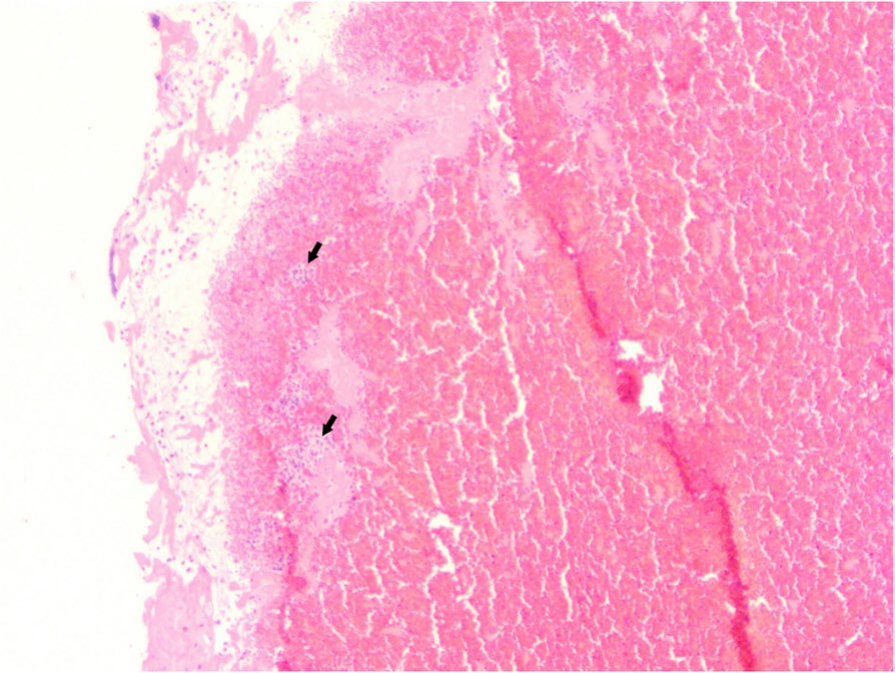
Histopathology of the removed thrombus which showed many neutrophils trapped within a fibrin network as well as many scattered nuclear debris (black arrowheads), suggestive of a strong inflammatory response

In addition to blood stasis, endothelial injury also plays an important role in PVS thrombosis. PV amputation during lobectomy injures the endothelium and consequently activates the extrinsic pathway of the coagulation cascade. Approximately 3% of patients who received anticoagulation therapy after left atrial appendage occlusion developed cerebral thromboembolisms [18, 19]. As the left atrium is connected to the systemic circulation, cardiac surgery can potentially trigger cerebral thromboembolism via the development of nonbacterial thrombotic endocarditis caused by endothelial injury. Lung surgery during transplantation can also trigger cerebral thromboembolism via thrombus formation in the PV [20]. Thus, endothelial injury per se may cause pulmonary venous thrombosis; no hemodynamic effect is in play [21]. Usui et al. reported a patient with a cerebral embolism developing after left lower lobectomy. Pathologically, the thrombus evidenced a large number of neutrophils, which suggests that an inflammatory response caused by vascular endothelial injury to the PVS was also significant in terms of thrombosis [21]. Indeed, in one of our cases, many neutrophils were evident in the thrombus (Fig. 2), which suggests the involvement of inflammation. Thus, both blood stasis and endothelial injury trigger PVS thrombus formation after lobectomy [4, 13, 20]; this thrombosis is thought to cause cerebral embolism.

### Treatment and prognosis

The incidence of perioperative stroke varies depending on the surgical population. The overall incidence of perioperative stroke in noncardiac, nonneurological surgical patients in the United States is approximately 0.1% based on multiple cohort studies [22, 23] and is approximately 0.4–0.6% in post-lobectomy patients [1, 2], which is slightly higher. Recent studies have reported that the incidence of cerebral infarction after left upper lobectomy is as high as 1.9–4.6% [4, 5]. Thus, clinicians should pay attention to these patients.

Mechanical thrombectomy is currently indicated for the management of AIS with LVO of the anterior circulation in patients for whom the procedure can be performed within 6 h of symptom onset, although some recent studies have extended patient selection to 24 h after onset if certain CT perfusion-based criteria are met [24, 25]. Such treatment is cost-effective for patients with AIS [26]. Thrombolysis is usually performed in addition to thrombectomy, but this is not possible in postsurgical populations. However, thrombectomy alone improves outcomes [27].

In our patients, the average time from onset to recanalization was 165.5 min. Nine patients (90%) had a good prognosis (mRS ≤ 2) at the 3-month follow-up. Therefore, timely recanalization of AIS patients with LVOs developing after pulmonary lobectomy can result in a good prognosis. Of our 10 patients, 8 underwent direct aspiration, but 2 (Nos. 4 and 5) required the use of a stent retriever because the occluded vessel was a small terminal branch. In our experience, direct aspiration had a high recanalization rate (100%) and was quick. This is our recommended method, and post-lobectomy patients with LVOs should be treated in institutions with thrombectomy capability. None of the patients developed a severe complication; three patients (Nos. 1, 2, and 10) exhibited asymptomatic hemorrhagic transformation after thrombectomy. Secondary anticoagulation therapy (rivaroxaban 20 mg per day) was prescribed for all patients after imaging (brain CT or magnetic resonance imaging) revealed no hemorrhage; we encountered no recurrent stroke during follow-up (average 13.7 months).

The limitations of our study were the small number of patients, the retrospective design of the analysis and the possible selection bias. The long-term outcomes of these patients were uncertain, and the duration of anticoagulation therapy for secondary prevention was unknown. These limitations reflect the need for further research in this area. A large, multicenter study is warranted for further study.

## Conclusion

We suggest that postoperative PVS thrombosis causes cerebral embolism. Endovascular therapy effectively treats AIS with LVO developing after lung surgery, and direct aspiration is a promising strategy. A team approach toward early recognition of stroke, diagnostic evaluation via multimodal CT, and prompt treatment is key when minimizing poor neurological outcomes. A large, multicenter study is warranted to further confirm these findings.

## Data Availability

The datasets generated and analysed during the current study are not publicly available due to data protection of the patients but are available from the corresponding author on reasonable request.
